# Maternal slaughter at abattoirs: history, causes, cases and the meat industry

**DOI:** 10.1186/2193-1801-2-125

**Published:** 2013-03-22

**Authors:** Peter Olutope Fayemi, Voster Muchenje

**Affiliations:** Department of Livestock and Pasture Science, University of Forte Hare, Private Bag X1314, Alice, Eastern Cape, 5700 South Africa

**Keywords:** Buller syndrome, Housekeeping pain genes, Livestock restocking, Meat branding, Non-coding microRNA (miRNAs), Pain biomarkers, Slaughter reforms

## Abstract

Animals of both sexes are slaughtered for meat and meat products at the abattoirs. It has been found in few countries that one-out-of-every-three ewe and one-out-of-every-four doe with single, twin or multiple foetuses are slaughtered in registered slaughterhouses. In quest for meat, numerous viable embryos and foetuses are wasted unnoticed since the productive pregnant animals are not spared in the process. The trend in the slaughter of pregnant animals for meat (ranging from 54.5% to 70.1%) therefore calls for a review to reminisce issues on slaughter reforms, emphasize its implication on losses of genetic materials and sustainability of meat production. As a way of ensuring that welfare quality® assessment is not compromised and cruelty is prevented during animal slaughter, the quantitation of housekeeping genes and naturally occurring microRNAs (miRNAs) are recommended for identifying candidate pain biomarkers. In order to respect consumers’ right however, the manuscript suggests meat branding where the consumption of meat from pregnant animals is ethical.

## Introduction

Livestock husbandry and slaughter have become millenary traditions in every part of the World. The main reason for these traditions is that humans are at the top of the biological chain in the habit of meat consumption (Okoli et al. 2006). This therefore serves as the foremost reason for maintaining animal populations to provide a nutritious and desirable form of food for people (Adama et al. 2011). Although globally, the practice of slaughtering different breeds of livestock has been sustained, the pregnancy status of the animal being slaughtered for meat still remains a hideous issue in many countries (Aberle et al. 2001 ; Warriss, 2008). The scenario of animal slaughter in abattoirs has shown that not only the conventional non-breeding livestock are slaughtered for meat but also the productive pregnant and lactating ones (Gregory and Grandin, 2007 ; Whitlock and Maxwell, 2008 ; Adama et al. 2011). These animals are either killed for daily meals or occasionally for rituals, religious festivals, ceremonies, drug formulations, disease control or to meet immediate financial needs (Gregory and Grandin 2007 ; Cadmus and Adesokan, 2010,2012).

 inspection (Grandin The attendant embryonic and foetal wastages due to this practice consequently query the efficiency of *ante-mortem*^2004^ ; Addass et al. 2010) and the rationale for converting pregnant animals to beef, mutton, chevon, pork, offal and other meat types. It also challenges the ethical evidence supporting the act of slaughtering pregnant animals in situations where there is no law supporting the practice. In most cases, meat consumers are even unaware or denied the right to know the status of the animal that is converted to meat they eat. In the past, studies have been focused on: animal right (Galvin and Herzog 1992), protection of animal welfare standards (Botreau et al. 2007 ; Thornber 2010), conservation of animals’ genetic resources (Woolliams et al. 2007); pre-slaughter stress responsiveness (Terlouw et al. 2008; Muchenje et al. 2009a); abattoir and slaughter surveillance (Addass et al. 2010), animal slaughter and meat quality (Hoffman et al. 2009 ; Muchenje et al. 2008; 2009b). Obviously, these studies have made novel contributions to animal welfare and meat science in general yet; cases of slaughtering pregnant animals for meat production have not received adequate attention. Hence, this review therefore attempts to: retrace history behind the promulgation of slaughter reforms; highlight causes and cases of maternal slaughter. It also suggests some ideas on how the quantitation of pain biomarkers can aid “humane slaughter of animals or Welfare Quality® assessment” for preventing cruelty to animals at slaughter, curbing the extinction of meat species and for respecting consumers’ right.

## Reminiscence on abattoir and slaughter reforms

The practice of animal slaughter to produce meat for consumption is inadvertently dated back to antiquity. The history behind the existence of public abattoirs had been traced to Roman civilization and France, between 15th and 16th centuries (Bello and Oyedemi, 2009). The law of 1890 in Italy required that public abattoirs be provided in all communities of more than six thousand inhabitants (Oldfield 1895). Similar developments were reported in Norway, Sweden, Denmark, Netherlands and Rumania (Jode et al. 1906). During the 1880’s and early 1890’s, the animal-protectionists, veterinarians and anti-Semitic societies in Saxony and in parts of Germany consequently lobbied for slaughterhouse reforms (Judd 2003). They sought the licensing of slaughterers and the restriction of the abattoir operations to men only. They also proposed the implementation of stricter inspection procedures and the stunning of animals into a state of unconsciousness before their slaughter. These groups as well, called for change because they were convinced that the current state of affairs in the municipally-run slaughter houses posed a risk to the public’s health (Judd 2003). In their view, the activities at the abattoir allegedly encouraged cruel behaviour, attracted unsavoury characters from employees and were responsible for the accumulation of contaminants from its dirty and bloody surfaces (Judd 2003 ; Ubwa et al. 2013).

Saxon animal protectionists also expressed concern with the traditional ways in which animals were slaughtered for food (Metcalf 1989 ; Judd 2003 ; Lavi 2007). The concern later became the Dutch Veterinarians’ motto: ‘*Hominum animaliumque saluti*, ‘to the benefit of man and animal alike’ indicating their role in maintaining animal resources and protecting animal and human health (Smith and Philips 2002). Laws for the protection of animals existed in the Australian states before Australia’s federation in 1901 (Jode et al. 1906) and remained the dominant policy instrument for animal welfare until the 1980s. These laws were directed against cruelty which was interpreted as willful or needless infliction of pain or spiteful neglect of the animal (Grandin 2004). While the prevention of cruelty to animal acts were revised regularly to keep pace with the time, it became apparent in the latter half of the twentieth century that these anti-cruelty acts provided insufficient protection for animal welfare (Thornber 2010). This is because the subject of animal slaughter and their pre-slaughter welfare is considered to be very unpleasant. Often, the personnel concerned prefer not to know the details of what goes on inside a slaughterhouse or rather feel secured to conceal it in order to avoid controversy or suspicion (Grandin 2004).

## Cases of slaughtering pregnant animals at the abattoirs

**Figure 1:**
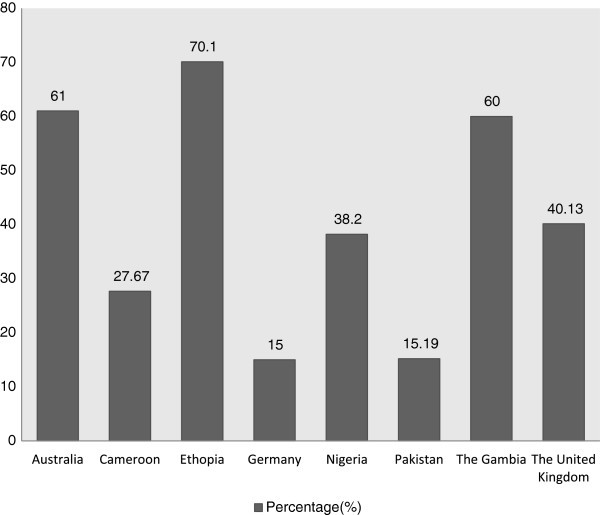
**Published cases on the conversion pregnant animals to meat.** Sources: Ernst (2009); Goosens et al. (1998); Gregory & Grandlin (2007); Khan & Khan (1989); Ladds et al. (2008); Muhammad et al. (2009); Mukasa-Mugerwa &Tekelye (2003); Riehn et al. (2010); Sanusi et al. (2006); Singleton (1996).

Ideally, sound economic livestock management, demands that animals sold for slaughter should be mainly males and reproductively inactive females (Opara et al. 2006 ; Abdulkadir et al. 2008 ; Cudworth 2008 ; Riehn et al. 2010). In various slaughterhouses nevertheless, cases of converting productive, clinically healthy livestock at different gestational stages into meat have been reported (Table 1 & Figure 1). A study on the Ethiopian Highland sheep showed that 70.1% ewes were found pregnant in the process of slaughter and 24% of them were with twins (Mukasa-Mugerwa and Tekelye, 2003). The record from a semi-arid abattoir in Nigeria, also confirmed that 34.3% (of the 0.26 million) ewes were pregnant at the point of slaughter (Muhammad et al. 2009). Earlier in the same region, out of 0.21 million goats that are slaughtered yearly, 26.1% of these ‘does’ were pregnant Sanusi et al. (2006). The result further showed that many pregnant cows were slaughtered (62%) for meat and more male foetuses (56.7%) were wasted than the female foetuses in the process Adama et al. (2011). Approximately 15% of the heifers in 53 German slaughterhouses attested to their pregnancy at the point of slaughter (Riehn et al. 2010).

**Table 1:** **Pregnancy stage and foetal number of slaughtered pregnant animals**

Animal	Pregnancy stage (1st -3rd Trimester)			Foetal number			Sources
	1st (%)	2nd (%)	3rd (%)	Single	Twin	Multiple	
Buffalo	NS*	NS*	NS*	Single	NS*	NS*	Khan and Khan (1989).
Carmel	NS*	2nd	NS*	NS*	NS*	NS*	Sonfada et al. (2009).
Cows	NS*	15.00	15-26.67	Single calf	90%	90%	Singleton (1996); Opara et al. (2006 ); Ernst, 2009 and Riehn et al. (2010).
Does	NS*	NS*	NS*	NS*	NS*	NS*	-
Mares	NS*	NS*	NS*	NS*	NS*	NS*	-
Sows	NS*	NS*	NS*	NS*	NS*	NS*	-
Ewes	NS*	NS*	NS*	NS*	24%	NS*	Mukasa-Mugerwa and Tekelye (2003).

NS*: Not Specified.

In all these cases, various pregnancy diagnoses have been adopted to ascertain the status of the meat species presented for slaughter. This is necessary as a means of conserving valuable genetic resources from livestock and consequently work towards sustainability of meat production (Ślósarz et al. 2007 ; Verberckmoes et al. 2004 ; Whitlock and Maxwell 2008 ; Grazul-Bilska et al. 2010) using pregnancy-associated glycoproteins as biomarkers (Garbayo et al. 2000 ; Jerome 2012). The presence of early conceptus proteins such as ovine Trophablast protein I (OTP-1) which prevents the release of PGF2α has also been used for pregnancy diagnosis in sheep (Bretzlaff and Romano 2001) and the appearance of cytokinen at approximately day 15 post mating as pregnancy indicator in goats (Goossens et al. 1998). Laparoscopy, laparotomy within 18–25 day post mating and serum progesterone (P4) values higher than 1 ng/ml; the secretion of 17& 22–24 KDa proteins on day 17 post mating in the caprine conceptus, non-return to eostrus and the expression of follistatin and activin during oestrous cycle confirmed follicular development at slaughter have been reported in literature too (Wani 1982 ; Xia et al. 2010).

More than 90% of the affected animals in different states of gravidity have also been found slaughtered during the last two trimesters of pregnancy. Among many techniques, several authors (Tajik et al. 2001 ; Boscos et al. 2003 ; Verberckmoes et al. 2004 ; Yotov 2007 have confirmed the pregnancy status at second trimester using: maternal serum alpha fetoprotein (MSAFP) levels; maternal serum chorionic gonadotropin levels (α hCG, beta hCG peak concentration); unconjugated estriol; inhibin (α and β-inhibins); multiple marker screening; neutrophils alkaline phosphatase; scwangeschafts protein1; the proform of eosinophil major basic protein (proMBP); placental isoferrintin p43 component; foetal cell sorting; bioactive modulators (E2 and F_2α_) and metabolizing enzymes for characterization of DNA^+.^ In their third trimester, palpation and ballotment; foetal measurements; ultrasonography and morphology imaging of endometrial have established the pregnancy status of cows, does, ewes, mares, sows and others (Beg et al. 2001 ; Bretzlaff and Romano 2001 ; Calamari 2001 ; Flores et al. 2001 ; Verberckmoes et al. 2004 ; Whitlock and Maxwell, 2008 ; Grazul-Bilska et al. 2010).

The visual assessment of the reproductive tracts from the right and left side and, the palpation of the uterus, ovaries and oviduct have been used at a local abattoir in Pakistan to substantiate that 61% of the cows were pregnant at the point of slaughter (Khan and Khan, 1989). In buffaloes and cows, each pregnancy was reported comprising a single foetus with the distribution of 51.11% on the right and 48.88% on left side in buffaloes whereas in cows, 57.14% on the right and 42.85% on left side (Khan and Khan 1989 ; Ladds et al. 2008). Harvested ovaries from the slaughtered animals have equally shown evidence of current and past reproductive states of such animals in the form of follicles at varying stages of oestrous cycles, embryonic trophoblastic adhesions, implantation and foetal-placental developments (Arthur et al. 1982 ; Bretzlaff and Romano 2001 ; Okoli et al. 2006).

## Socio-economic and physiologic reasons for converting pregnant animals to meat

**Figure 2:**
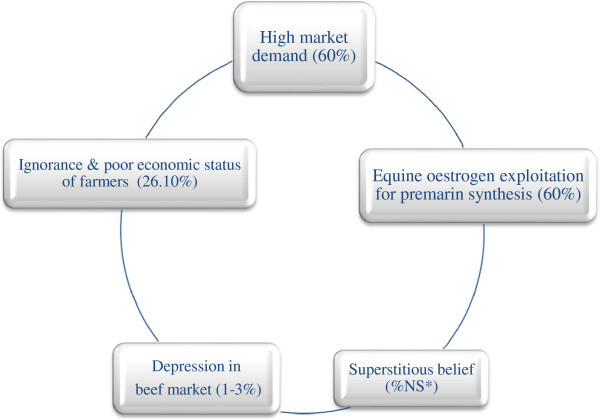
**Socio-economic motives for culling and slaughtering pregnant livestock at the abattoirs.** Key: NS*means Not Specified. Sources: Creidi et al. (2005); Frick et al. (2002); Gregory & Grandlin (2007); Muhammad et al. (2007); Riehn et al. (2010); Sanusi et al. (2006); Williams (1994).

Literature has shown that the wastage of the conceptus through haphazard slaughter of pregnant livestock is one of the practices man has ever used against his production endeavour (Garba et al. 1998 ; Umar et al. 2006; Bello et al. 2008). This has been observed as a key factor responsible for protein malnutrition in some African countries and a possible constraint to future livestock populations in the continent (Nwakpu and Osakwe 2007 ; Ademola 2010 ; Cadmus and Adesokan 2010). On the contrary, the act of maternal slaughter (in most cases) tends to frustrate the efforts of breeders, geneticists and nutritionists as it poses the risk of widening the gap of animal protein requirements by meat consumers (Khan and Khan 1989). Poor financial condition of the farmers and ignorance of the pregnancy state of the animals (Figure 2) were the reasons advanced for culling and slaughtering pregnant livestock (Sanusi et al. 2006 ; Muhammad et al. 2009).

The exploitation of conjugated equine oestrogen (CEE) from pregnant horses for the treatment of menopausal syndrome in women is another reason behind the slaughter of pregnant horses for meat. The utilization of this hormone for alleviating age-related mnemonic decline or for improving spatial reference memory and for the prevention of scopolamine-induced amnesia is connected with the reason for massive slaughter of pregnant horses (Frick et al. 2002 ; Creidi et al. 2005). The extraction of Premarin from pregnant horse to enhance the facial skin of ageing post-menopausal women also support, the biochemical benefits for the slaughter of pregnant animals in some countries (Williams 1994 ; Frick et al. 2002).

The recurrence of spontaneous abortion by pregnant heifers within 3 weeks of arrival at the feedlots has been noted by some farmers as a rationale for presenting their pregnant heifers for slaughter. The disruptive bulling behaviour being recognised was implicated by other farmers for slaughtering pregnant cows by Gregory and Grandlin (2007). The occurrence of bulling amounting to 11% during the peak period in autumn and winter has always left the victimized in-calf cows ill as a result of unwanted mounting by the buller steers. The exhibition of buller syndrome by the bulling steers usually influences some cattle farmers in the USA to cull their injured pregnant cows. The depressions in the beef market in the United State of America have sometimes compelled farmers as well to offer 1–3% of their pregnant heifers for slaughter and retain fewer heifers on their farms as breeding replacements (Gregory and Grandin 2007). It was reported that a total of 28.29% of cows in their third trimester of pregnancy were culled because of infertility, 21.8% for mastitis and 15.8% as a result of old age (Singleton 1996 ; Ernst 2009).

Approximately, 27.3% of the farmers who consigned these animals for slaughter were ignorant of the fact that the cows were actually pregnant but only 21.8% of the farmers were aware of the physiological status of these cows. At least 6% of the culled cows were “discarded” for fertility reasons when they were actually pregnant. Otherwise known as euthanasia, few authors have indicated that emergency slaughter may be an exceptional situation that might necessitate slaughtering pregnant animals (Grandin 1994 ; Butterworth, 2000 ; Biggs and Blackwell 2005). When emergency slaughter remains the only alternative, it is expected that the farmer contact the slaughter operators and the Official Veterinarian must rather be present during the *ante-mortem* and *post-mortem* inspection to declare the meat either fit or unfit for human consumption (Gregory and Grandin 2007). This is the only permissible situation in the USA when maternal slaughter will not be considered as cruelty to the animal (Singleton 2010) otherwise; fines will be paid by the culprit (Perera 2006 ; Cowan 2012).

In the USA and Sri Lanka, a sum of one hundred thousand Sri Lankan Rupee (Rs 100,000.00; which is equivalent to USD 888.54) is paid as fine in Sri Lanka for violating the revolutionary legislation on cruelty to animals (Perera 2006). The guilty abattoir operator in the USA goes through chains of punishments. The license of the convicted abattoir operator will either be suspended or revoked for conviction of a felony and offense on animal cruelty. A sum of one thousand US Dollar is paid for the first offense, five thousand USD for the second and ten thousand USD for the third and subsequent offenses for cruelty on livestock including maternal slaughter of animals at slaughterhouses (Laura 2010). The United States of America also has a version of H.R.2744 (USDA’s FY2006) appropriation bill to prohibit non-ambulatory livestock (also called downers) from being slaughtered for human food (Cowan 2012).

## Conclusion and recommendation on maternal slaughter

Non-conformity to the rules for which only unproductive, infertile, sterile, old or accidentally injured animals are allowed to be slaughtered shows as a drift from the original code of conduct on public abattoir operation (Jode et al. 1906 ; Judd 2003). So far from pre-slaughter welfare perspective, no information is available on pain manifestations and anoxic signals that accompany the production of cytotoxic cascade and activation of brain damaging processes when pregnant animals are slaughtered. Due to human preference for veal, a study has shown that calf foetuses do not feel pains when electrically stunned based on the impact of endogenous neuron-inhibitors Mellor et al. (2005); Mellor (2010). Hitherto, there is no report indicating if the same is obtainable when pregnant cows, ewes, mares, does, sows and others are stunned before exanguination. Through indigenous knowledge system (IKS) where animals are slaughtered without stunning, dearth of information still exists even when pregnant animals, calves, lambs or kids are conducted through traditional slaughter proceedings (Fayemi and Muchenje 2012).

As postulated in cellular genomics (Bartel 2004 ; Moqil 2012), there is a possibility that studies on the roles of naturally occurring non-coding micro ribonucleic acids (miRNAs) might profoundly inhibit the expression of pain genes and epigenetic mechanisms. Since miRNAs are endogenously produced, its molecular mechanism fuels the binding of small-interfering RNA strands to Argonaute proteins to form effectors complexes known as RNA induced silencing complex. In animals, miRNAs function in a way to direct mRNA cleavage or repress translation of complementary RNAs during brain development, organogenesis, pathways signalling, apoptosis, metabolism, cardiogenesis and many other biological processes (McDaneld 2009 ; Wang et al. 2010). It becomes expedient in future studies therefore, to link the roles of endogenous neuron-inhibitors with changes in noiceptors miRNA producing enzymes while pregnant meat species undergo stunning and slaughter processes at the abattoir or in traditional settings.

Moreover, research efforts should be directed to use appropriate pain biomarkers at different parity stages to characterize response to slaughter pains (with or without stunning) by pregnant animals. Molecular quantification of neuropathic biomarkers (ubiquitin C-terminal hydrolase-L1; S-100β neuroprotein; glial fibrillary acidic protein; neuron specific enolase, etc.) will be necessary to ascertain the degree of gene disorder or analgesic sensitivity of pregnant animals at slaughter. The characteristic expressions of housekeeping genes are also suggested as means of understanding syndromes featuring absence or presence of relevant pain candidate genes in pregnant animals at slaughter. More studies should also be focused on:

- The breed of livestock that is more prone to maternal slaughter and subtle erosion of genetic materials.
- Restocking programme of meat species commonly slaughtered when pregnant.
- Kind of pregnancy (normal or ectopic) and foetal physiological condition from livestock that are presented for slaughter.
- Diet -related risk factor or diet intervention related to the consumption of meat from pregnant animals.
- Creation of consumer awareness and branding of meat from pregnant animals.
